# Recent Advances in Oral Squamous Cell Carcinoma

**DOI:** 10.3390/jcm11216406

**Published:** 2022-10-29

**Authors:** Ana Caruntu, Constantin Caruntu

**Affiliations:** 1 Department of Oral and Maxillofacial Surgery, “Carol Davila” Central Military Emergency Hospital, 010825 Bucharest, Romania; 2 Department of Oral and Maxillofacial Surgery, Faculty of Dental Medicine, “Titu Maiorescu” University, 031593 Bucharest, Romania; 3 Department of Physiology, “Carol Davila” University of Medicine and Pharmacy, 050474 Bucharest, Romania; 4 Department of Dermatology, “Prof. N.C. Paulescu” National Institute of Diabetes, Nutrition and Metabolic Diseases, 011233 Bucharest, Romania

Oral squamous cell carcinoma (OSCC), the most frequent of head and neck cancers, has been a topic of great interest to the scientific community. This is due to both its very high incidence and mortality rates in many countries around the world, as well as its complex social and economic impacts for patients that manage to survive this highly disabling disease [1]. The paradox about OSCC is the simplicity to diagnose the disease, with suspicion being aroused after a routine oral examination in most cases, and the overwhelming number of patients that are diagnosed in advanced stages. The impact of a late diagnosis in OSCC is dramatic in terms of prognosis, quality of life for the patients and the financial burden for healthcare systems worldwide [2]. In recent decades, there have been crucial breakthroughs in the therapeutic management of OSCC. One example is the introduction of free flap reconstruction techniques, starting with the “Chinese flap” first reported in 80s for large tumors previously considered unresectable, which allowed the extension of the therapeutical armamentarium in OSCC [3]. Immunotherapy that was recently registered for advanced and metastatic head and neck cancers is another example of efficient strategy to combat OSCC [4]. However, despite all these efforts, the overall prognosis of OSCC has failed to significantly improve in recent decades. In the incipient stages of OSCC, the reported survival rates after treatment completion can exceed 90% [5], and clinical experience shows that almost half of patients have advanced disease at the moment of diagnosis [6,7]; therefore, it is logical that the most important aspect in the combat against OSCC is a timely detection of the disease. Furthermore, since risk factors induce disease in most patients [8], efficient prevention strategies are vital in the global efforts to fight against OSCC. Consequently, in recent years, many investigations have addressed these particular aspects of OSSC.

Screening strategies in OSCC, currently based on oral clinical examination, have not changed survival rates in recent decades [9]. By contrast, prevention campaigns against smoking and alcohol abuse, as well as recently introduced HPV vaccination campaigns proved to be highly efficient strategies, which are continuously changing the pattern of incidence and risk-factor-associated OSCC rates [10]. Another important objective is to identify and diagnose premalignant lesions that develop long before the malignant transformation occurs or even to identify subjects at high risk of developing an oral cancer. High-resolution imaging techniques, in particular in vivo reflectance confocal microscopy (RCM), have been investigated for the discrimination of premalignant lesions from malignancies through a non-invasive, reproducible method and is currently used in clinical practice [11]. Regarding specific morphological features described in RCM images, alteration of the typical epithelial honeycomb pattern and the presence of target keratinocytes in the epidermis are common findings in premalignant lesions, such as actinic cheilitis. Malignant transformation in suspicious non-healing mucosal wounds is suggested when a complete epidermal disarray occurs in these patients [12]. The focus is currently oriented towards defining reliable RCM diagnostic criteria for many types of epithelial diseases that can reduce the indication of conventional diagnostic biopsy [13].

As in many other malignancies, complex metabolic alterations have been reported in OSCC that can be graphically detected through imaging techniques. High glucose uptake, which is characteristic for malignant behavior, has been suggested as a potential screening method for oral cancer using the imaging technique of 18F- fluorodeoxyglucose positron emission tomography/computed tomography (18F-FDG PET/CT), which is currently used for diagnosis, staging and monitoring in OSCC. Recent data provide strong evidence for the utility of PET-CT in cancer screening. This method is associated with improved long-term outcomes in asymptomatic patients [14]. Furthermore, Ishikawa et al. reported that in OSCC, salivary metabolites revealed significant correlations with the maximum standardized uptake value, suggesting a novel potential approach for the early detection of oral cancer [15].

Imaging techniques have reached an unimaginable level of quality and versatility compared to several decades ago, and modern medicine greatly relies on imaging in both diagnosis and treatment planning in most diseases, including OSCC. In recent years, an increasing interest has been oriented towards artificial intelligence for a fast and accurate diagnosis based on imaging investigations. Shavlokhova et al. conducted an interesting study assessing the utility of deep learning processes on the interpretation of ex vivo fluorescent confocal microscopy images for the diagnosis of OSCC. Although the study sample is relatively low, preliminary results suggest that there might be a paradigm changing the potential implementation of artificial intelligence for the pathological diagnosis of OSCC [16].

In the quest for non-invasive diagnostic tools, saliva has been proposed as an alternative for the early detection, diagnosis and monitoring of OSCC and oral premalignant lesions. Babiuch et al. investigated the immunological mechanisms in oral carcinogenesis, expressed through the alterations of proinflammatory NF-kappaB dependent cytokines in premalignant and malignant oral mucosa. Their results identified IL-8 as the most relevant biomarker for the malignant transformation in OSCC, with changes detectable both in tumor tissue and salivary samples [17]. Furthermore, the salivary levels of other proinflammatory cytokines, such as IL-6 and TNF-alfa, were also increased in OSCC patients compared to healthy controls or patients with oral dysplastic lesions, thus confirming the important role of chronic inflammation in the pathogenesis of OSCC. Genetic alterations in OSCC have also been investigated through salivary determinations, revealing prognostic features. Oh et al. reported important salivary mARN changes for a series of six genes that exhibited significantly lower expressions in OSCC saliva samples. Assessed in combination, monoamine oxidase B and NGFI-A binding protein 2 were an important predictive element for the early detection of OSCC in younger patients [18]. These findings provide an important insight into the pathogenetic mechanisms of OSCC, and further investigations are required to confirm the biomarker potential of mRNA though salivary determinations in OSCC.

In addition to the early detection in OSCC, an important aspect is the accuracy of prognostic stratification, especially in patients with advanced disease. Nodal involvement is a common finding at presentation in OSCC, and the prognosis dramatically changes in these patients. A recent study conducted by Shimomura et al. reported an increased expression of the non-structural maintenance of chromosome (SMC) Condensin I Complex Subunit H (NCAPH) in patients with OSCC in correlation with regional lymphatic spread [19]. Furthermore, NCAPH was associated with resistance to chemotherapy and worse prognosis in these patients. These findings imply underlying mechanisms between NCAPH alterations and OSCC pathogenesis. Unveiling these mechanisms might provide new therapeutic strategies in the future for OSCC patients. In addition to lymphatic spread, many clinical studies confirmed the negative predictive role of extranodal extension in head and neck cancer [20], but the main challenge is the detection of this feature, which currently implies pathology examination after surgical removal of the lymph nodes as part of neck dissection. Tsai et al. identified significant metabolomic alterations in tumor tissue of patients with advanced OSCC and extranodal extension, which can be detected in plasma, thus implying the predictive character of the metabolomic alterations for extranodal extension in OSCC [21].

Interestingly, even though it is known that OSCC, alongside other types of upper aero-digestive tract malignancies, is more common in the elderly population, it seems that age does not significantly impact cancer-related prognosis [22]. Many studies investigated the aspects related to age and cancer-related survival. Onishi et al. reported that the only independent predictive factor in elderly patients diagnosed with esophageal squamous cell carcinoma was sarcopenic obesity [23]. The main challenge is related to the difficulties in diagnosing sarcopenic obesity based on clinical and imaging findings, which limits the implementation of this method into clinical practice.

There are many similarities between OSCC and cutaneous squamous cell carcinoma (cSCC) in terms of pathogenetic mechanisms [24]. Many types of genetic alterations have been associated with progression to both OSCC and cSCC. Genes involved in cell cycle control, epithelial cell differentiation, epithelial-to-mesenchymal transition, cell survival, in addition to the inactivation of tumor suppressor genes, TP53 being most frequently reported, are currently considered the drivers for progression to malignancy [25]. Recent discoveries highlighted promising features in non-coding RNA molecules—miRNAs and lncRNAs—which could interfere with carcinogenetic processes in the post-transcriptional stage as potential therapeutic targets in many types of cancer, including epithelial malignancies, and represent a popular topic in the scientific community [25]. Pre-existing premalignant lesions, namely actinic keratosis (AC), are a common finding in cSCC, and cellular plasticity is an essential feature for progression to malignancy. This epithelial-to-mesenchymal transition was manipulated in experimental models, and the results reveal that zinc finger E-box binding homeobox 1 (ZEB1), one of the EMT markers, upregulated in cSCC samples, attained an increased expression in A431 squamous cell carcinoma cell line after the knockdown of ovo-like zinc finger-2 (OVOL2), facilitating an aggressive behavior [26]. These pathogenetic mechanisms could be further developed and might be proposed in the future for the prevention of cSCC.

In conclusion, the data presented above emphasize the magnitude and impact of squamous cell carcinomas worldwide and the increased interest of the scientific community to elucidate the molecular mechanisms that lead to malignancy and cause their aggressive behavior. In the quest for novel, accessible diagnostic strategies and therapeutic targets in squamous cell carcinoma, promising elements have been identified, which could be developed into effective treatment strategies that will improve the prognosis for all patients affected by this malignancy.

## References

[1] https://seer.cancer.gov/statfacts/html/oralcav.html.

[2] Grafton-Clarke C., Chen K.W., Wilcock J. (2019). Diagnosis and Referral Delays in Primary Care for Oral Squamous Cell Cancer: A Systematic Review. Br. J. Gen. Pract..

[3] Steel B.J., Cope M.R. (2015). A Brief History of Vascularized Free Flaps in the Oral and Maxillofacial Region. J. Maxillofac. Surg. Off. J. Am. Assoc. Oral Maxillofac. Surg..

[4] Caruntu A., Scheau C., Tampa M., Georgescu S.R., Caruntu C., Tanase C. (2021). Complex Interaction Among Immune, Inflammatory, and Carcinogenic Mechanisms in the Head and Neck Squamous Cell Carcinoma. Adv. Exp. Med. Biol.-Clin. Exp. Biomed..

[5] https://www.cancer.org/cancer/oral-cavity-and-oropharyngeal-cancer/detection-diagnosis-staging/survival-rates.html.

[6] Thompson-Harvey A., Yetukuri M., Hansen A.R., Simpson M.C., Adjei Boakye E., Varvares M.A., Osazuwa-Peters N. (2020). Rising Incidence of Late-Stage Head and Neck Cancer in the United States. Cancer.

[7] Jou A., Hess J. (2017). Epidemiology and Molecular Biology of Head and Neck Cancer. Oncol. Res. Treat..

[8] Chi A.C., Day T.A., Neville B.W. (2015). Oral Cavity and Oropharyngeal Squamous Cell Carcinoma-an Update. CA Cancer J. Clin..

[9] Ghantous Y., Abu Elnaaj I. (2017). Global Incidence and risk factors of oral cancer. Harefuah.

[10] Salehiniya H., Raei M. (2020). Oral Cavity and Lip Cancer in the World: An Epidemiological Review. Biomed. Res. Ther..

[11] Lupu M., Popa I.M., Voiculescu V.M., Caruntu A., Caruntu C. (2019). A Systematic Review and Meta-Analysis of the Accuracy of in VivoReflectance Confocal Microscopy for the Diagnosis of Primary Basal Cell Carcinoma. J. Clin. Med..

[12] Lupu M., Caruntu A., Boda D., Caruntu C. (2020). In Vivo Reflectance Confocal Microscopy-Diagnostic Criteria for Actinic Cheilitis and Squamous Cell Carcinoma of the Lip. J. Clin. Med..

[13] Hofmann-Wellenhof R., Pellacani G., Malvehy J., Soyer H.P. (2012). Reflectance Confocal Microscopy for Skin Diseases.

[14] Chan H.-P., Liu W.-S., Liou W.-S., Hu C., Chiu Y.-L., Peng N.-J. (2020). Comparison of FDG-PET/CT for Cancer Detection in Populations with Different Risks of Underlying Malignancy. In Vivo.

[15] Ishikawa S., Hiraka T., Kirii K., Sugimoto M., Shimamoto H., Sugano A., Kitabatake K., Toyoguchi Y., Kanoto M., Nemoto K. (2020). Relationship between Standard Uptake Values of Positron Emission Tomography/Computed Tomography and Salivary Metabolites in Oral Cancer: A Pilot Study. J. Clin. Med..

[16] Shavlokhova V., Sandhu S., Flechtenmacher C., Koveshazi I., Neumeier F., Padrón-Laso V., Jonke Ž., Saravi B., Vollmer M., Vollmer A. (2021). Deep Learning on Oral Squamous Cell Carcinoma Ex Vivo Fluorescent Confocal Microscopy Data: A Feasibility Study. J. Clin. Med..

[17] Babiuch K., Kuśnierz-Cabala B., Kęsek B., Okoń K., Darczuk D., Chomyszyn-Gajewska M. (2020). Evaluation of Proinflammatory, Nf-Kappab Dependent Cytokines: Il-1α, Il-6, Il-8, and TNF-α in Tissue Specimens and Saliva of Patients with Oral Squamous Cell Carcinoma and Oral Potentially Malignant Disorders. J. Clin. Med..

[18] Oh S.Y., Kang S.M., Kang S.H., Lee H.J., Kwon T.G., Kim J.W., Lee S.T., Choi S.Y., Hong S.H. (2020). Potential Salivary MRNA Biomarkers for Early Detection of Oral Cancer. J. Clin. Med..

[19] Shimomura H., Sasahira T., Nakashima C., Kurihara-Shimomura M., Kirita T. (2020). Non-Smc Condensin i Complex Subunit h (Ncaph) Is Associated with Lymphangiogenesis and Drug Resistance in Oral Squamous Cell Carcinoma. J. Clin. Med..

[20] Huang S.H., Chernock R., O’Sullivan B., Fakhry C. (2021). Assessment Criteria and Clinical Implications of Extranodal Extension in Head and Neck Cancer. Am. Soc. Clin. Oncol. Educ. Book.

[21] Tsai C.K., Lin C.Y., Kang C.J., Liao C.T., Wang W.L., Chiang M.H., Yen T.C., Lin G. (2020). Nuclear Magnetic Resonance Metabolomics Biomarkers for Identifying High Risk Patients with Extranodal Extension in Oral Squamous Cell Carcinoma. J. Clin. Med..

[22] Malik A., Mishra A., Chopda P., Singhvi H., Nair S., Nair D., Laskar S.G., Prabhash K., Agarwal J.P., Chaturvedi P. (2019). Impact of Age on Elderly Patients with Oral Cancer. Eur. Arch. Oto-Rhino-Laryngol..

[23] Onishi S., Tajika M., Tanaka T., Yamada K., Abe T., Higaki E., Hosoi T., Inaba Y., Muro K., Shimizu M. (2020). Prognostic Impact of Sarcopenic Obesity after Neoadjuvant Chemotherapy Followed by Surgery in Elderly Patients with Esophageal Squamous Cell Carcinoma. J. Clin. Med..

[24] Georgescu S.R., Tampa M., Mitran C.I., Mitran M.I., Caruntu C., Caruntu A., Lupu M., Matei C., Constantin C., Neagu M. (2020). Tumor Microenvironment in Skin Carcinogenesis. Tumor Microenvironments in Organs.

[25] Lazar A.D., Dinescu S., Costache M. (2020). Deciphering the Molecular Landscape of Cutaneous Squamous Cell Carcinoma for Better Diagnosis and Treatment. J. Clin. Med..

[26] Murata M., Ito T., Tanaka Y., Yamamura K., Furue K., Furue M. (2020). OVOL2-Mediated ZEB1 Downregulation May Prevent Promotion of Actinic Keratosis to Cutaneous Squamous Cell Carcinoma. J. Clin. Med..

